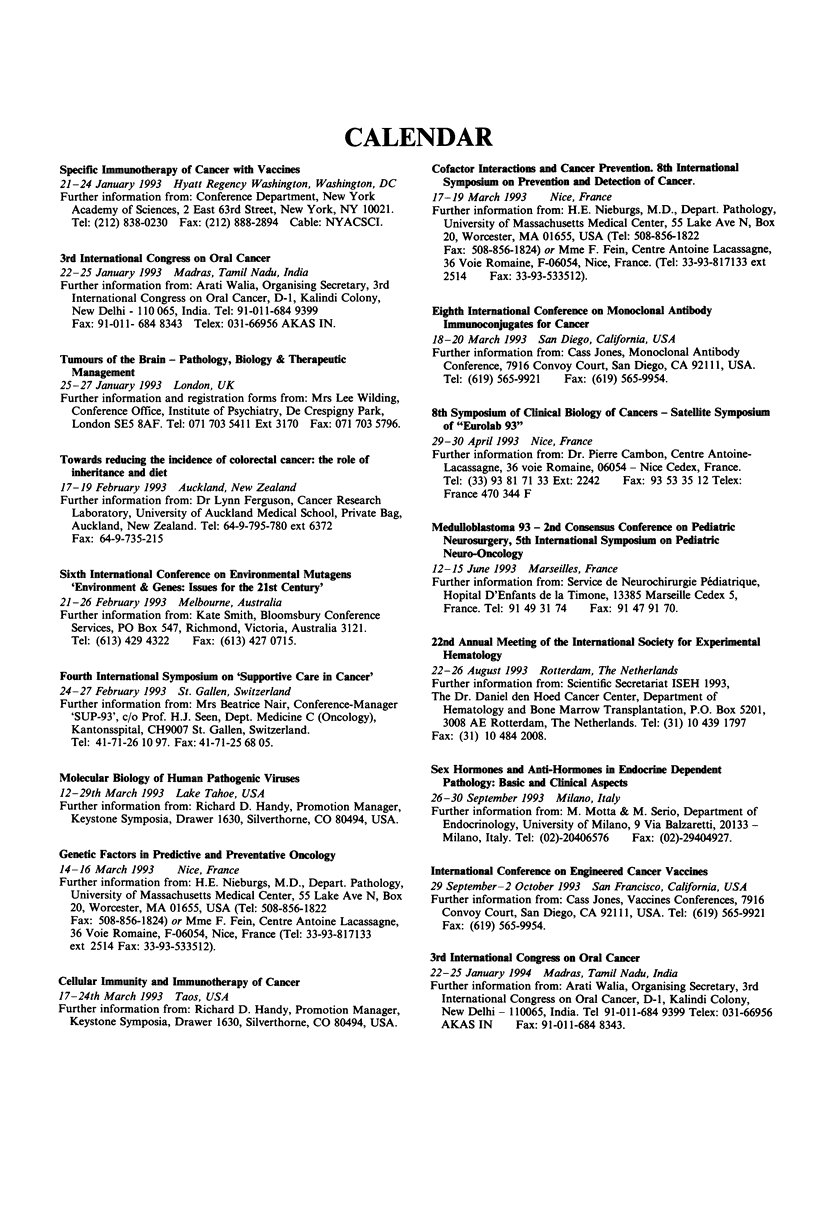# Calendar

**Published:** 1992-12

**Authors:** 


### CALENDAR

**Specific Immunotherapy of Cancer with Vaccines**
*21–24 January 1993 Hyatt Regency Washington, Washington, DC*
Further information from: Conference Department, New York Academy of Sciences, 2 East 63rd Street, New York, NY 10021. Tel: (212) 838-0230 Fax: (212) 888-2894 Cable: NYACSCI.

**3rd International Congress on Oral Cancer**
*22–25 January 1993 Madras, Tamil Nadu, India*
Further information from: Arati Walia, Organising Secretary, 3rd International Congress on Oral Cancer, D-1, Kalindi Colony, New Delhi - 110 065, India. Tel: 91-011-684 9399 Fax: 91-011- 684 8343 Telex: 031-66956 AKAS IN.

**Tumours of the Brain – Pathology, Biology & Therapeutic Management**
*25–27 January 1993 London, UK*
Further information and registration forms from: Mrs Lee Wilding, Conference Office, Institute of Psychiatry, De Crespigny Park, London SE5 8AF. Tel: 071 703 5411 Ext 3170 Fax: 071 703 5796.

**Towards reducing the incidence of colorectal cancer: the role of inheritance and diet**
*17–19 February 1993 Auckland, New Zealand*
Further information from: Dr Lynn Ferguson, Cancer Research Laboratory, University of Auckland Medical School, Private Bag, Auckland, New Zealand. Tel: 64-9-795-780 ext 6372 Fax: 64-9-735-215

**Sixth International Conference on Environmental Mutagens 'Environment & Genes: Issues for the 21st Century'**
*21–26 February 1993 Melbourne, Australia*
Further information from: Kate Smith, Bloomsbury Conference Services, PO Box 547, Richmond, Victoria, Australia 3121. Tel: (613) 429 4322 Fax: (613) 427 0715.

**Fourth International Symposium on 'Supportive Care in Cancer'**
*24–27 February 1993 St. Gallen, Switzerland*
Further information from: Mrs Beatrice Nair, Conference-Manager 'SUP-93', c/o Prof. H.J. Seen, Dept. Medicine C (Oncology), Kantonsspital, CH9007 St. Gallen, Switzerland. Tel: 41-71-26 10 97. Fax: 41-71-25 68 05.

**Molecular Biology of Human Pathogenic Viruses**
*12–29th March 1993 Lake Tahoe, USA*
Further information from: Richard D. Handy, Promotion Manager, Keystone Symposia, Drawer 1630, Silverthorne, CO 80494, USA.

**Genetic Factors in Predictive and Preventative Oncology**
*14–16 March 1993 Nice, France*
Further information from: H.E. Nieburgs, M.D., Depart. Pathology, University of Massachusetts Medical Center, 55 Lake Ave N, Box 20, Worcester, MA 01655, USA (Tel: 508-856-1822 Fax: 508-856-1824) *or* Mme F. Fein, Centre Antoine Lacassagne, 36 Voie Romaine, F-06054, Nice, France (Tel: 33-93-817133 ext 2514 Fax: 33-93-533512).

**Cellular Immunity and Immunotherapy of Cancer**
*17–24th March 1993 Taos, USA*
Further information from: Richard D. Handy, Promotion Manager, Keystone Symposia, Drawer 1630, Silverthorne, CO 80494, USA.

**Cofactor Interactions and Cancer Prevention. 8th International Symposium on Prevention and Detection of Cancer.**
*17–19 March 1993 Nice, France*
Further information from: H.E. Nieburgs, M.D., Depart. Pathology, University of Massachusetts Medical Center, 55 Lake Ave N, Box 20, Worcester, MA 01655, USA (Tel: 508-856-1822 Fax: 508-856-1824) *or* Mme F. Fein, Centre Antoine Lacassagne, 36 Voie Romaine, F-06054, Nice, France. (Tel: 33-93-817133 ext 2514 Fax: 33-93-533512).

**Eighth International Conference on Monoclonal Antibody Immunoconjugates for Cancer**
*18–20 March 1993 San Diego, California, USA*
Further information from: Cass Jones, Monoclonal Antibody Conference, 7916 Convoy Court, San Diego, CA 92111, USA. Tel: (619) 565-9921 Fax: (619) 565-9954.

**8th Symposium of Clinical Biology of Cancers – Satellite Symposium of "Eurolab 93"**
*29–30 April 1993 Nice, France*
Further information from: Dr. Pierre Cambon, Centre Antoine-Lacassagne, 36 voie Romaine, 06054 – Nice Cedex, France. Tel: (33) 93 81 71 33 Ext: 2242 Fax: 93 53 35 12 Telex: France 470 344 F

**Medulloblastoma 93 – 2nd Consensus Conference on Pediatric Neurosurgery, 5th International Symposium on Pediatric Neuro-Oncology**
*12–15 June 1993 Marseilles, France*
Further information from: Service de Neurochirurgie Pédiatrique, Hopital D'Enfants de la Timone, 13385 Marseille Cedex 5, France. Tel: 91 49 31 74 Fax: 91 47 91 70.

**22nd Annual Meeting of the International Society for Experimental Hematology**
*22–26 August 1993 Rotterdam, The Netherlands*
Further information from: Scientific Secretariat ISEH 1993, The Dr. Daniel den Hoed Cancer Center, Department of Hematology and Bone Marrow Transplantation, P.O. Box 5201, 3008 AE Rotterdam, The Netherlands. Tel: (31) 10 439 1797 Fax: (31) 10 484 2008.

**Sex Hormones and Anti-Hormones in Endocrine Dependent Pathology: Basic and Clinical Aspects**
*26–30 September 1993 Milano, Italy*
Further information from: M. Motta & M. Serio, Department of Endocrinology, University of Milano, 9 Via Balzaretti, 20133 – Milano, Italy. Tel: (02)-20406576 Fax: (02)-29404927.

**International Conference on Engineered Cancer Vaccines**
*29 September–2 October 1993 San Francisco, California, USA*
Further information from: Cass Jones, Vaccines Conferences, 7916 Convoy Court, San Diego, CA 92111, USA. Tel: (619) 565-9921 Fax: (619) 565-9954.

**3rd International Congress on Oral Cancer**
*22–25 January 1994 Madras, Tamil Nadu, India*
Further information from: Arati Walia, Organising Secretary, 3rd International Congress on Oral Cancer, D-1, Kalindi Colony, New Delhi – 110065, India. Tel 91-011-684 9399 Telex: 031-66956 AKAS IN Fax: 91-011-684 8343.